# *Prevotella melaninogenica* Alleviate *Mycoplasma pneumoniae* Infection Through the Butyrate Based on Multi‐Omic Analysis and Experimental Validation

**DOI:** 10.1111/cbdd.70397

**Published:** 2026-09-06

**Authors:** Zhengyang Xue, Huiliang Xu, Ling Zhu, DeYu Zhao

**Affiliations:** ^1^ Department of Respiratory Medicine Children's Hospital Affiliated to Nanjing Medical University Nanjing China; ^2^ Department of Respiratory Medicine Affiliated Children's Hospital of Jiangnan University, Wuxi Children's Hospital Wuxi China; ^3^ Department of Respiratory Medicine Affiliated Changzhou Children's Hospital of Nantong University Changzhou China

**Keywords:** metabolic networks and pathways, metagenome, *Mycoplasma pneumoniae* pneumonia, pulmonary microbiota, respiratory tract infections, transcriptome

## Abstract

*Mycoplasma pneumoniae* (MP) is one of the main pathogens causing atypical pneumonia in children. The susceptible population is mainly children and adolescents over 5 years old, and the infection rate has increased in recent years. At present, there is limited research on the pulmonary microbiota of patients with *Mycoplasma pneumoniae* pneumonia, and the characteristics of their microbiota are not yet clear. We included MPP children in stages and established two independent cohorts. Cohort I (*n* = 175) performed 16S rRNA sequencing on bronchoalveolar lavage fluid (BALF) to explore microbial genus level characteristics, while Cohort II (*n* = 41) performed metagenomic and transcriptome sequencing to explore microbial species level characteristics and predict inter group differential metabolic pathways. Finally, a murine model infected with MP was established to validate the effects of *Prevotella melaninogenica* and its metabolite butyrate. Based on Multi‐Omic Analysis, we discovered that *P. melaninogenica* was the most discriminative species enriched in the critically ill group. Functional profiling demonstrated that butanoate metabolism pathways were significantly enriched in the severe group and positively correlated with *P. melaninogenica* abundance. Transcriptomic analysis revealed that *P. melaninogenica* ‐associated host genes were significantly enriched in immune regulation pathways. Animal experiments confirmed that both *P. melaninogenica* and butyrate pretreatment significantly attenuated MP‐induced pulmonary inflammation, pathogen load, and immune cell infiltration. Respiratory microbiota dysbiosis may be associated with MPP severity. *Prevotella melaninogenica* , a potential protective commensal enriched in severe group MPP patients, may alleviate airway inflammation through its metabolite butyrate.

## Graphical abstract

At the genus level, Prevotella abundance is negatively associated with disease severity, further pinpointed at the species level that *Prevotella melaninogenica* serves as the core effector species and validated the anti‐inflammatory function of its metabolite butyrate. The genus *Prevotella* overall exhibits a protective phenotype, with *P. melaninogenica* playing a key role as the core effector species.

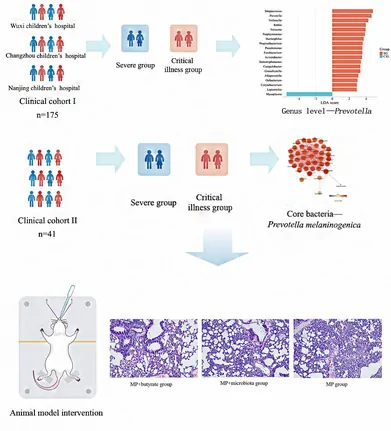

## 1 Introduction

*Mycoplasma pneumoniae pneumonia* (MPP) is a common community‐acquired pneumonia. This disease is caused by *Mycoplasma pneumoniae* (MP) infection and has been prevalent several times in recent years, affecting the health of children worldwide (Tang et al. 2025). In China, *Mycoplasma pneumoniae* infections occur throughout the year, with a seasonal peak typically observed in autumn and winter. From October to December 2023, the MP infection rate rapidly increased in China, with a positive rate of 33.56% for *Mycoplasma pneumoniae* in acute respiratory infections in November (Oishi and Ouchi 2022; Sun et al. 2025). The prevalence of this disease has brought a significant economic burden to families, society, and the country.

As a common commensal in upper and lower respiratory tract (Tett et al. 2021), *Prevotella* species help to assist the internal environment of the body to maintain stability when the body is healthy or during an infection. There are many types of *Prevotella*, and different subspecies have different effects in different diseases or healthy states. However, accumulating evidence has implicated *Prevotella* enrichment in various inflammatory disorders, including periodontitis, rheumatoid arthritis, and bacterial vaginosis (Larsen 2017). In respiratory infections, the precise role of *Prevotella* remains controversial, with some studies suggesting protective effects and others proposing pro‐inflammatory functions (Klug et al. 2020; Horn et al. 2022). Within this genus, *Prevotella melaninogenica* represents a species of particular interest, given its prevalence in the respiratory tract and its reported association with inflammatory conditions (Tett et al. 2021). Butyrate (Wang et al. 2025; Yu et al. 2025), primarily produced by Firmicutes and certain Bacteroidetes including some *Prevotella* species, has well‐documented anti‐inflammatory properties through multiple mechanisms (Chen et al. 2024). These findings point to a potential “microbiota‐metabolite‐immune” axis in MPP pathogenesis, with *Prevotella* species potentially serving as key modulators of this axis.

However, the integration of multiple omics layers—microbiome, metabolome, and host transcriptome—to construct a comprehensive mechanistic model has been rarely attempted in MPP research (Horn et al. 2022; Chen et al. 2021; Wei et al. 2024, 2026; Yan et al. 2022; Ashizawa et al. 2025). In this study, we integrated clinical specimen microbiota analysis (16S rRNA sequencing and shotgun metagenomics), host transcriptomic profiling, and animal model interventions to investigate the impact of airway microbiota and the metabolite butyrate on MP infection. We focused primarily on the genus *Prevotella* as a key microbial modulator of disease severity, with secondary focus on the species *Prevotella melaninogenica* as a representative candidate for in‐depth mechanistic investigation.

## 2 Methods

### 2.1 Recruitment and Clinical Information of Pediatric Patients

Cohort 1 consisted of pediatric MPP patients who received fiberoptic bronchoscopy treatment at the Respiratory Departments of three tertiary hospitals (NanJing Children's Hospital Affiliated to NanJing Medical University, ChangZhou Children's Hospital, and WuXi Children's Hospital) from March to August 2023.

Cohort 2 enrolled children diagnosed with *Mycoplasma pneumoniae* pneumonia (MPP) who underwent fiberoptic bronchoscopy at the Department of Respiratory Medicine, Nanjing Medical University Children's Hospital, between January and December 2021.

The definition of mycoplasma infection is determined based on the highest read in tNGS or the relative abundance.

This study was approved by the institutional ethics committees: Ethics Committee of Nanjing Children's Hospital (approval numbers: 202012089‐1, 202211213‐1), Ethics Committee of Wuxi Children's Hospital (approval numbers: WXCH2022‐12‐106), Ethics Committee of Changzhou Children's Hospital (approval numbers: 2023‐018). This study was conducted in accordance with the Declaration of Helsinki. Authors had no access to information that could identify individual participants during or after data collection. This study has obtained written informed consent from the guardians of the participants to participate in this study.

### 2.2 Inclusion and Exclusion Criteria

#### 2.2.1 Inclusion Criteria

(1) Presence of fever and respiratory symptoms/signs; (2) Pneumonia confirmed by chest X‐ray or pulmonary CT; (3) Positive MP‐DNA or RNA. (4)Consistent with anyone of the following indications for bronchoscopy surgery: (A) Recurrent respiratory infections(More than 5 times per year); (B) Abnormal chest imaging: Atelectasis; (C) Diagnosis and treatment of pulmonary infectious diseases.

#### 2.2.2 Severity Classification

If there is persistent high fever, no improvement in imaging findings, or life‐threatening severe extrapulmonary complications such as sepsis and infectious shock, which require mechanical ventilation or other life‐support treatments, the case is classified as the critical illness group. All other cases are categorized as the severe group.

#### 2.2.3 Exclusion Criteria

(1) Underlying chronic diseases (e.g., immunodeficiency, congenital heart disease); (2) Immunosuppressive therapy within 3 months.

### 2.3 Microbial Sequencing of Lung Microbiota

#### 2.3.1 16S rRNA Sequencing (Cohort I)

When performing fiberoptic bronchoscopy on a child, collect BALF samples. During sampling, repeated saline irrigation was performed and the aspirated fluid was stored in the reagent tube. When submitting for testing, the second tube was taken for testing to prevent infection from the upper respiratory tract flora. Store these samples in a −80°C refrigerator within 4 h after sampling. Next, genomic DNA was extracted and electrophoresis was performed on 1% agarose gel. Subsequently, qualified DNA samples were subjected to PCR amplification targeting the 16S rRNA gene, followed by fluorescence quantification, library construction, and sequencing on the Illumina Miseq platform. The obtained dual end reads are merged based on overlapping parts, followed by quality control and filtering. After sample deduplication, sequence clustering (or denoising) and classification identification were performed.

#### 2.3.2 Metagenomic Sequencing (Cohort II)

Genomic DNA was extracted using the QIAamp DNA Stool Mini kit, following the manufacturer's instructions. The concentration and purity of the DNA were measured using the Qubit quantitative system and agarose gel electrophoresis. Whole‐genome metagenomic sequencing was performed on the Illumina NovaSeq platform.

### 2.4 Transcriptome Sequencing (Cohort II)

Total RNA was extracted from the samples. The purity and integrity of the RNA were determined by agarose gel electrophoresis and Nalase spectrophotometer (OD260/280 ≥ 1.8 and RIN value ≥ 7). The library construction used oligodeoxycytidine magnetic beads to enrich mRNA with poly(A) tails, followed by random lysis and cDNA synthesis using random primers. The cDNA ends were repaired and ligated with sequencing adapters. The library was immobilized on flow cells and cluster generation was performed through bridge PCR amplification. Sequencing was conducted using fluorescent‐labeled ddNTPs and detected by laser scanning.

### 2.5 Animals Experiment Design

Specific pathogen‐free (SPF) male C57BL/6 mice, aged 6–8 weeks, weighed 18–20 g, were purchased from the Comparative Medicine Center of Yangzhou University. All operations were conducted in an experimental environment approved by the Institutional Animal Ethics Committee, and the operators received training on animal euthanasia procedures. These procedures have been carried out in accordance with applicable veterinary guidelines (China Veterinary Medical Association).

All animals were housed in an SPF facility with a constant temperature and a 12‐h light/dark cycle, with free access to food and water. After 1 week of acclimatization, the mice were randomly divided into three groups (*n* = 6 per group): the MP control group, the MP + microbiota group, and the MP + butyrate group. From day 0 to day 2, mice in the MP + microbiota and MP + butyrate groups received intratracheal administration for three consecutive days. The MP + microbiota group received *Prevotella melaninogenica* suspension (30 μL/mouse, 1 × 10^9^ CFU), while the MP + butyrate group received a sodium butyrate solution (200 mg/kg). On Day 3, all mice were inoculated with *Mycoplasma pneumoniae* (MP) using the same intratracheal route (100 μL/mouse, 1 × 10^7^ CFU) to establish the infection model. On Day 7, the mice were anesthetized, and a tracheostomy was performed for intubation. Bronchoalveolar lavage fluid (BALF) was collected by washing the lungs three times with an appropriate volume of sterile phosphate‐buffered saline (PBS). The BALF was centrifuged, and the supernatant was stored at −80°C for subsequent analysis. After BALF collection, the mice were euthanized, and the lung tissues were harvested and fixed in 4% paraformaldehyde for further histological examination. Cell count was determined using the Giemsa staining method, inflammatory factors were measured using the Elisa kit, and pathogen load was measured using qPCR.

### 2.6 Statistical Analysis

All statistical analyses were performed using SPSS software (version 19.0) and R software (version 4.0.3). Graphical visualizations were generated using the ggplot2 package (version 3.3.5) in R. A two‐tailed *p*‐value < 0.05 was considered statistically significant unless otherwise specified. Continuous variables were first tested for normality using the Shapiro–Wilk test. Normally distributed data were presented as mean ± standard deviation (SD) and compared between two groups using Student's *t*‐test. Non‐normally distributed data were expressed as median with interquartile range (IQR) and compared using the Mann–Whitney *U* test. Categorical variables were presented as counts and percentages (*n*, %) and compared using the chi‐square test or Fisher's exact test, as appropriate. Clinical parameters compared between severity groups included demographic characteristics, signs/symptoms, laboratory markers.

## 3 Results

### 3.1 Clinical Characteristics

In Cohort I, there were no significant differences in age (6.69 ± 2.84 years vs. 5.94 ± 2.22 years, *p* = 0.126) and sex distribution (male 46.6% vs. 64.3%, *p* = 0.204) between the severe and critical illness groups. In addition to the aforementioned findings, we observed that respiratory rate (RR) was significantly higher in the critical group than in the severe group (*p* < 0.05), while blood oxygen saturation without oxygen inhalation upon admission (SpO_2_) and lymphocyte count (L) were significantly higher in the severe group than in the critical group (all *p* < 0.05). No significant differences were observed in the remaining parameters (Table 1).

**TABLE 1:** Comparison of basic information between two groups of patients in Cohort I.

Characteristics	Total (*N* = 175)	Severe group (*n* = 91)	Critical illness group (*n* = 84)	*t*/*Z*/*χ* ^2^	*p*
Gender					
Male	85 (48.6)	43 (47.3)	42 (50.0)	1.617	0.204
Female	90 (51.4)	48 (52.7)	42 (50.0)		
Age (year)	6.55 ± 2.83	6.69 ± 2.84	5.94 ± 2.22	0.948^a^	0.126
Signs/symptoms					
Fever	166 (94.9)	82 (90.1)	81 (96.4)	0.825	0.364
Cough	174 (99.4)	89 (97.8)	83 (98.8)	0.087	0.767
Chest pain	3 (1.7)	3 (1.9)	0 (0.0)	0.265	0.606
RR (times/min)	24.00 (22.00, 27.00)	24.00 (22.00, 26.00)	28.00 (25.75, 33.00)	−3.194^b^	**0.001**
SpO_2_ (%)	98.00 (97.00, 98.50)	98.00 (97.00, 98.50)	94.70 (91.53, 97.00)	−3.873^b^	**< 0.001**
Blood routine					
CRP (mg/L)	10.22 (2.66, 26.00)	10.22 (2.57, 26.15)	10.15 (5.62, 25.58)	−0.424^b^	0.672
WBC (×10^9^/L)	8.20 (6.29, 10.57)	8.17 (6.41, 10.39)	10.47 (5.76, 16.22)	−1.545^b^	0.122
N (×10^9^/L)	6.77 (4.45, 57.80)	6.50 (4.45, 58.55)	9.20 (5.67, 15.37)	−0.126^b^	0.899
L (×10^9^/L)	3.35 (1.84, 22.1)	3.51 (1.91, 22.45)	1.65 (1.11, 2.61)	−3.119^b^	**0.002**
HB (g/L)	123.00 (119.00, 131.00)	124.00 (119.00, 131.00)	122.00 (115.25, 126.50)	−1.070^b^	0.284
PLT (×10^9^/L)	266.00 (209.00, 374.00)	265.00 (208.50, 374.00)	288.50 (212.25, 415.00)	−0.514^b^	0.607

*Note:* Bold font indicates statistical significance (*p* < 0.05).

Abbreviations: CRP, C‐reactive protein; HB, hemoglobin; L, lymphocyte count; N, neutrophil count; PLT, platelet count; RR, admission respiratory rate; SpO_2_, blood oxygen saturation without oxygen inhalation upon admission; WBC, white blood cell count.

^a^ *t* value.

^b^ *Z* value, others are *χ* ^2^ value; data are expressed as mean ± standard deviation (SD) for normally distributed continuous variables, median (interquartile range, IQR) for non‐normally distributed continuous variables, and number (percentage) for categorical variables. IQR is defined as the 25th and 75th.

In Cohort II, there were no significant differences in age (6.62 ± 2.93 years vs. 7.30 ± 2.75 years, *p* = 0.450) and sex distribution (male 52.4% vs. 25.0%, *p* = 0.072) between the severe and critical groups. In addition to the aforementioned findings, we observed that respiratory rate (RR) was significantly higher in the critical group than in the severe group (*p* < 0.05). Additionally, neutrophil count (N) and C‐reactive protein (CRP) levels were also significantly elevated in the critical group compared to the severe group (both *p* < 0.05). Conversely, blood oxygen saturation without oxygen inhalation upon admission (SpO_2_) was significantly lower in the critical group than in the severe group (*p* < 0.05). No significant differences were observed in the remaining parameters (Table 2).

**TABLE 2:** Comparison of basic information between two groups of patients in Cohort II.

Characteristics	Total (*N* = 41)	Severe group (*n* = 23)	Critical illness group (*n* = 18)	*t*/*Z*/*χ* ^*2*^	*p*
Gender					
Male	16 (39.0)	12 (52.2)	4 (22.2)	3.228	0.072
Female	25 (61.0)	11 (47.8)	14 (77.8)		
Age(year)	6.55 ± 2.83	6.62 ± 2.93	7.30 ± 2.75	−0.763^a^	0.450
Signs/symptoms					
Fever	39 (95.1)	20 (86.9)	18 (100.0)	0.635	0.467
Cough	40 (97.5)	20 (86.9)	18 (100.0)	0.067	0.897
Chest pain	1 (2.5)	0 (0.0)	1 (5.0)	0.096	0.756
RR (times/min)	24.50 (22.00, 27.00)	24.00 (22.00, 26.00)	27.75 (25.00, 33.00)	−3.456^b^	**< 0.001**
SpO_2_ (%)	98.00 (96.00, 99.00)	97.00 (96.00, 98.50)	93.75 (91.70, 96.00)	−3.696^b^	**< 0.001**
Blood routine					
CRP (mg/L)	10 (2.83, 29.00)	2.83 (2.83, 11.00)	15.00 (7.46, 34.50)	5.467^b^	**0.043**
WBC (×10^9^/L)	8.75 (6.69, 12.02)	8.54 (6.69, 10.66)	10.08 (6.68, 12.83)	0.605^b^	0.642
N (×10^9^/L)	68.50 (59.20, 76.10)	59.90 (52.10, 68.40)	73.75 (69.10, 78.45)	12.897^b^	**< 0.001**
PMN (×10^9^/L)	5.77 (4.29, 8.07)	4.92 (4.20, 7.16)	6.91 (4.65, 9.66)	4.111^b^	0.086
HB (g/L)	124.00 (120.00, 132.00)	124.00 (120.00, 132.00)	124.00 (118.75, 131.25)	0.023^b^	0.873
PLT (×10^9^/L)	279.00 (233.00, 350.00)	278.0 (233.00, 339.00)	282.50 (230.00, 353.25)	0.023^b^	0.873

*Note:* Bold font indicates statistical significance (*p* < 0.05).

Abbreviations: CRP, C‐reactive protein; HB, hemoglobin; L, lymphocyte count; N, neutrophil count; PLT, platelet count; RR, admission respiratory rate; SpO_2_, blood oxygen saturation without oxygen inhalation upon admission; WBC, white blood cell count.

^a^ Means *t* value.

^b^ Means *Z* value, others are *χ* ^*2*^ value; data are expressed as mean ± standard deviation (SD) for normally distributed continuous variables, median (interquartile range, IQR) for non‐normally distributed continuous variables, and number (percentage) for categorical variables. IQR is defined as the 25th and 75th.

### 3.2 The Distribution of Microbial Communities Varies Between Groups, With *Prevotella* Being a Significantly Different Bacterium Between Groups

**FIGURE 1:**
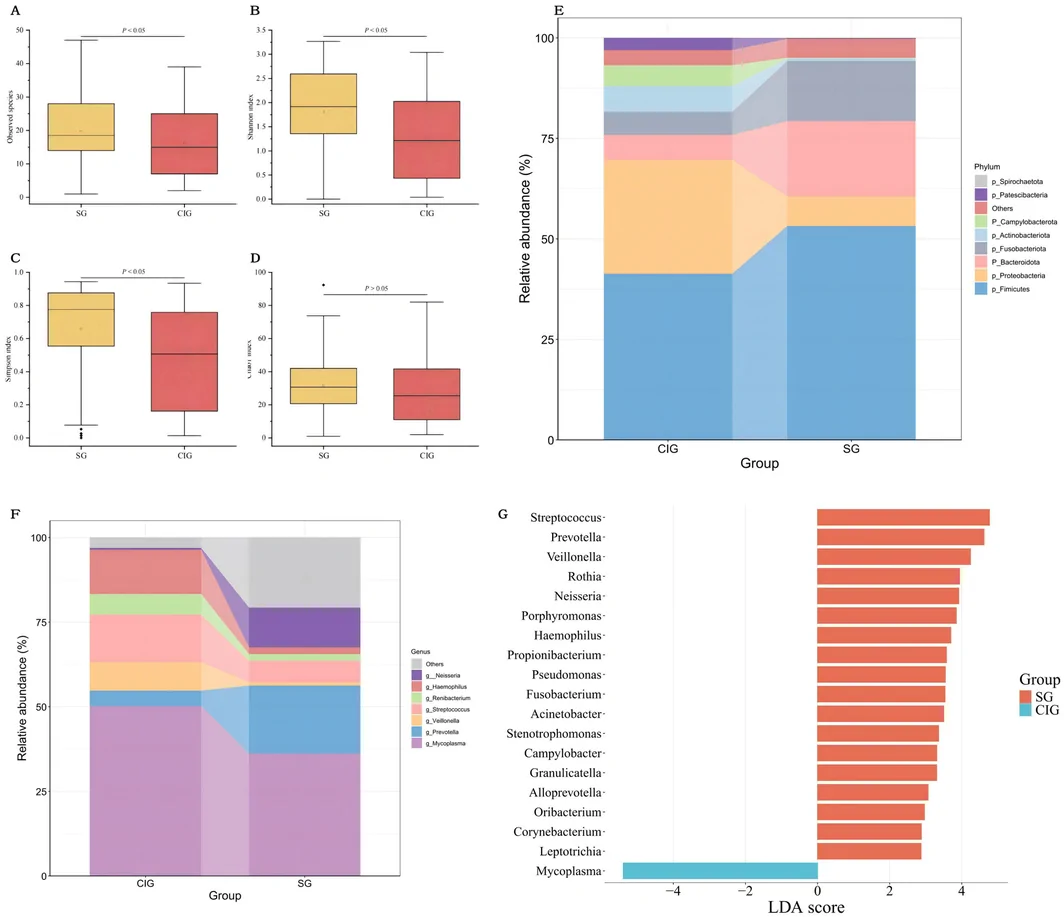
The genus *Prevotella* is an intergroup differential bacteria. Bronchoalveolar lavage fluid samples from 175 individuals in Cohort I were analyzed using 16S rRNA sequencing to compare the microbial community structures between the severe group (*n* = 91) and the critical group (*n* = 84). (A–D) Alpha diversity indices, including Observed species (A), Shannon index (B), Chao1 index (C), and Simpson index (D). Data are presented as mean ± SD. Statistical comparisons were performed using the Mann–Whitney U test. (E) Stacked bar plot showing the relative abundance of bacterial phyla in the two groups. (F) Stacked bar plot at the genus level showing the relative abundance of bacterial genera. (G) Linear discriminant analysis Effect Size (LEfSe) was employed to identify taxa with significant differential abundance between the two groups (LDA score > 2.5, *p* < 0.05). The bar plot displays the LDA scores for the most differentially abundant genera.

Alpha diversity analysis revealed that the Observed species, Shannon index, Chao1 index, and Simpson index of the severe group (Mann–Whitney *U* test, all *p* < 0.05 except Chao1 index, Figure 1A–D). Observation of the stacked bar plot at the phylum level revealed that Firmicutes, Proteobacteria, and Bacteroidetes were the predominant phyla common to both groups (Figure 1E). At the genus level, the stacked bar plot showed that the critical group exhibited a higher relative abundance of *mycoplasma* and *streptococcus*, whereas the abundance of *prevotella* was lower in this group (Figure 1F). To identify specific taxa driving this separation, we performed Linear discriminant analysis Effect Size (LEfSe) (LDA score > 2.5, *p* < 0.05). As shown in the LDA score bar plot (Figure 1G), several genera were differentially abundant. While *Streptococcus* exhibited the highest LDA score (LDA = 4.81), its clinical relevance was considered limited due to its high prevalence as an oral commensal and lack of specificity in severe pneumonia. Therefore, we focused on *Prevotella*, which had the second highest LDA score, LDA = 4.63 (except *mycoplasma*). *Prevotella* was significantly enriched in the severe group compared to the critical group, marking it as a phylotype of interest for subsequent validation.

### 3.3 *Prevotella melaninogenica* Is the First Differentially Expressed Bacterium at the Species Level, Associated With the Butyric Acid Metabolism Pathway

**FIGURE 2:**
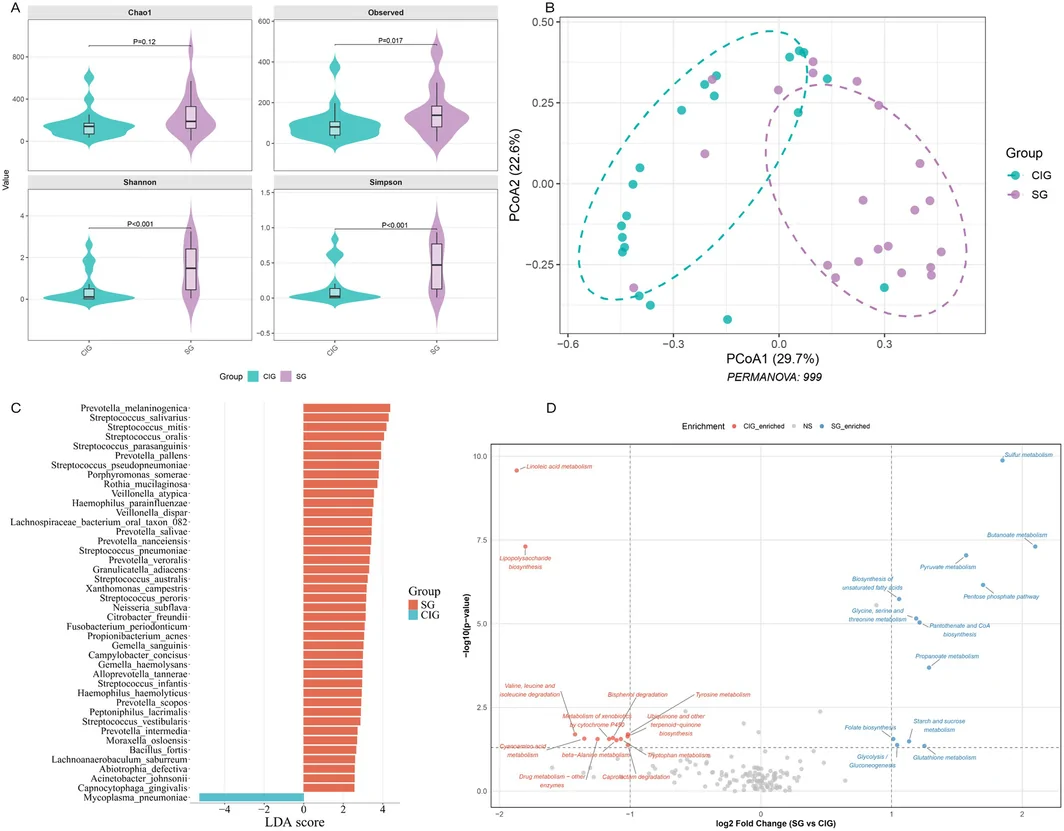
*Prevotella melaninogenica* is the first differentially expressed bacterial species between groups. Shotgun metagenomic sequencing was performed on bronchoalveolar lavage fluid samples from 41 individuals in Cohort II to validate and extend the findings from Cohort I at the species level. (A) Alpha diversity indices (Observed species, Shannon index, Chao1 index, and Simpson index) comparing the critical illness group (*n* = 18) and the severe group (*n* = 23). Data are presented as mean ± SD. Statistical comparisons were performed using the Wilcoxon test. (B) Beta diversity analysis based on metagenomic species profiles. Principal coordinate analysis (PCoA) plot showing the separation between the critical and severe groups. (C) Linear discriminant analysis Effect Size (LEfSe) identified differentially abundant species between the two groups (LDA score > 2.5, *p* < 0.05). The bar plot displays the LDA scores for the 41 most differentially abundant taxa. (D) Metabolic pathway profiling using HUMAnN3. Volcano plot visualizing differentially abundant metabolic pathways between the two groups (Differential expression analysis was conducted with thresholds of |log_2_ fold change| > 1 and adjusted *p*‐value < 0.05.). Pathways significantly enriched in the severe groupare highlighted in blue, while those enriched in the critical group are shown in red.

Remarkably, *prevotella melaninogenica* (belonging to Genus *prevotella*) emerged as the most discriminative feature (except *mycoplasma pneumoniae* ), with the highest LDA score (LDA = 4.22, Figure 2C). We performed metabolic pathway profiling using HUMAnN3. A total of 24 metabolic pathways were differentially abundant between the two groups (|log_2_ fold change| > 1 and adjusted *p*‐value < 0.05.), as visualized in the volcano plot (Figure 2D). Alpha diversity analysis revealed that species diversity (Observed species, Shannon index, Chao1 index and Simpson index) in the critical group was significantly lower than that in the severe group (all *p* < 0.05 except chao1), which was consistent with the observations from Cohort I (Figure 2A). Furthermore, beta diversity analysis based on metagenomic species profiles confirmed a significant separation between the critical group (*n* = 18) and the severe group (*n* = 23) (*R* ^2^ = 0.139, *p* < 0.05, Figure 2B). LEfSe analysis at the species level identified 42 differentiallbolism associated with *Prevotella* were significantly enriched in the severe group compared to the critical illness group.

**FIGURE 3:**
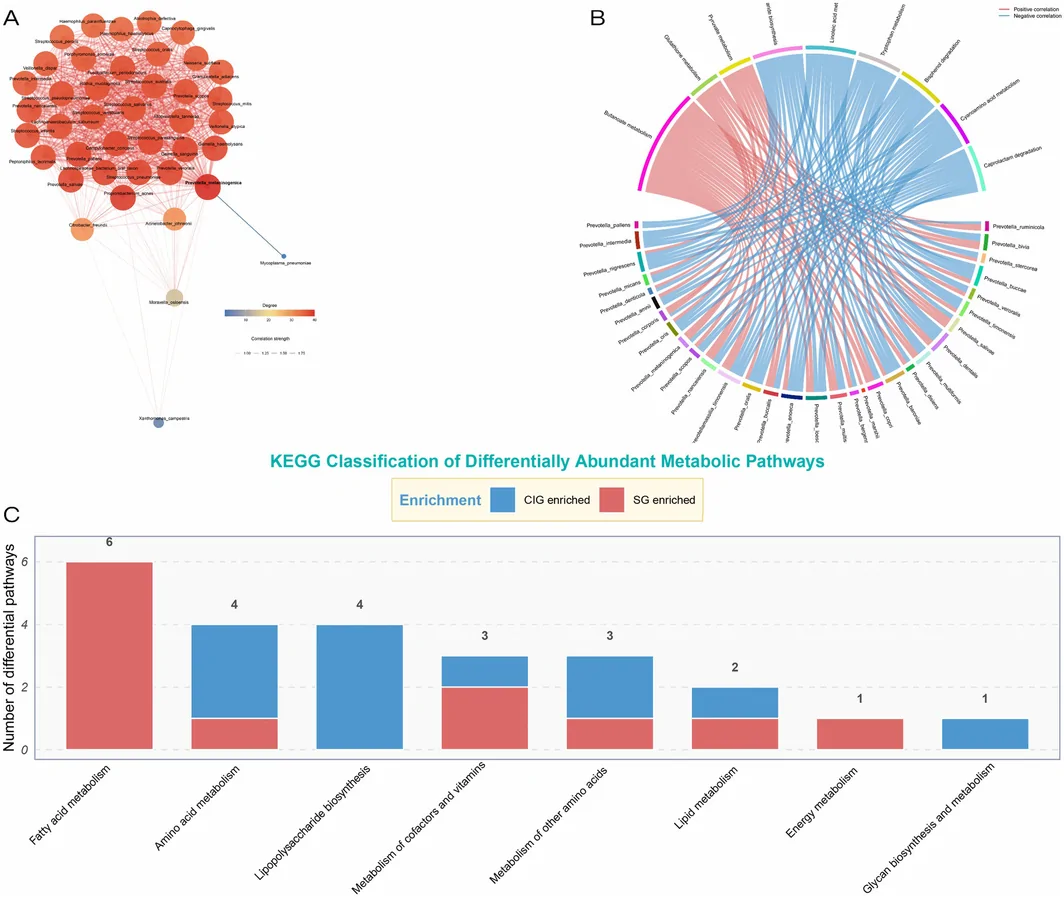
The abundance of *Prevotella melaninogenica* is related to inflammation expression and butyric acid metabolism pathway. (A) Co‐occurrence network analysis based on the 41 differentially abundant species identified by LEfSe. Each node represents a microbial species, node colors ranging from blue to red represent increasing degree centrality, while red and blue edges indicate positive and negative correlations, respectively, with edge thickness reflecting the correlation strength. (B) Chord diagram illustrating the correlations between *P. melaninogenica* abundance and differentially abundant metabolic pathways identified by HUMAnN3. Ribbon width represents the strength of Spearman's correlation. (C) The bar plot shows the distribution of significantly differential metabolic pathways (|log_2_FC| > 1, adjusted *p* < 0.05) across KEGG BRITE functional categories. The *x*‐axis represents the KEGG pathway categories, and the *y*‐axis indicates the number of pathways. Red and blue stacked bars represent pathways enriched in the critical illness and severe groups, respectively. Numbers above the bars indicate the total count of differential pathways in each category.

We next constructed a co‐occurrence network to investigate the ecological relationships among the 41 differential species. As shown in Figure 3A, *Prevotella melaninogenica* emerged as the most central hub node (degree = 40) and exhibited a negative correlation with *Mycoplasma pneumoniae* (Spearman's |ρ| = 0.65, *p* < 0.05). To link the microbiota to functional potential, we performed correlation analysis between the abundance of all *Prevotella* species and the 24 differentially abundant metabolic pathways identified earlier. As shown in the chord diagram (Figure 3B), the abundance of *Prevotella* species exhibited significant correlations with multiple metabolic pathways. Notably, the abundance of most *Prevotella* species was significantly positively correlated with the butanoate metabolism pathway (*p* < 0.05). Furthermore, the abundance of the majority of *Prevotella* species also showed significant positive correlations with several other metabolic pathways, including pyruvate metabolism (*p* < 0.05), glutathione metabolism (*p* < 0.05), and pantothenate and CoA biosynthesis (*p* < 0.05). Conversely, *Prevotella abundance* was significantly negatively correlated with multiple pro‐inflammation‐related pathways, including lipopolysaccharide biosynthesis (*p* < 0.05), linoleic acid metabolism (*p* < 0.05), and tryptophan metabolism (*p* < 0.05). As shown in Figure 3C, the differential pathways were distributed across multiple metabolic categories, with distinct patterns of enrichment between the groups.

### 3.4 The Decrease of *Prevotella melaninogenica* Exacerbating Pulmonary Inflammation

**FIGURE 4:**
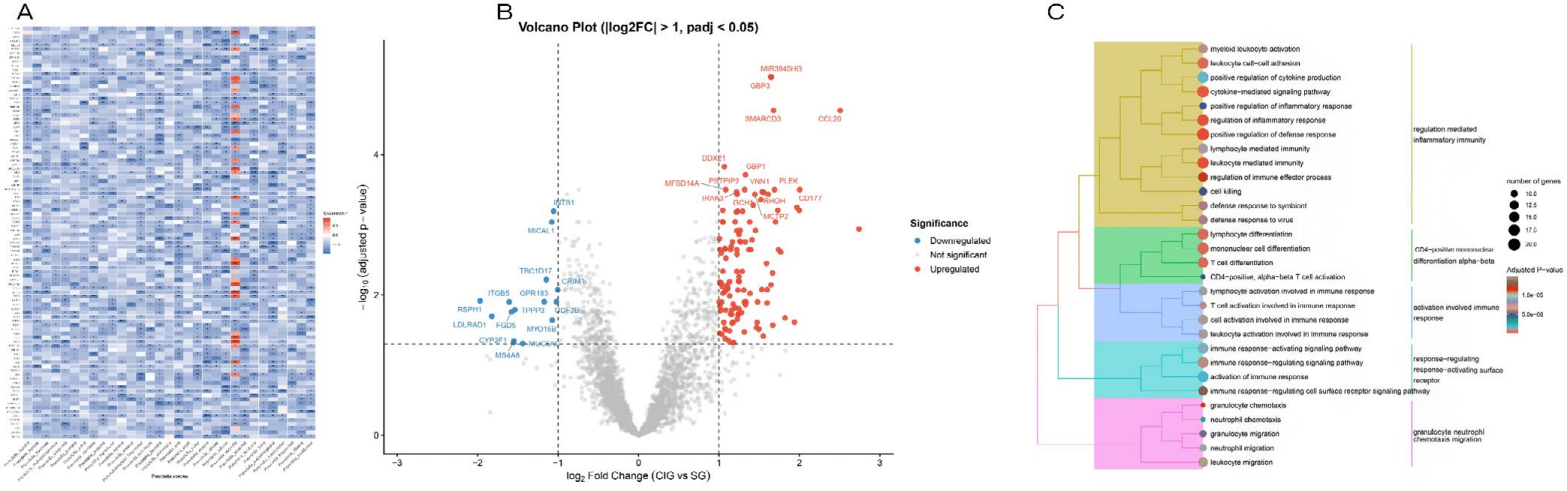
Integration of host transcriptomic responses with microbial and metabolic features. (A) Heatmap displaying the expression profiles of the top 100 host genes most strongly correlated with the abundance of at least one Prevotella species. Spearman correlation analysis was performed to identify genes significantly correlated with *Prevotella* abundance (|correlation coefficient| > 0.6, *p* < 0.05). (B) Volcano plot illustrating differentially expressed host genes between the critical illness and severe groups, restricted to the Prevotella‐associated gene set identified. Differential expression analysis was conducted with thresholds of |log_2_fold change| > 1 and adjusted *p*‐value < 0.05. (C) Presents a treeplot of GO biological process enrichment for overlapping genes significantly correlated with Prevotella abundance and differentially expressed between the critical illness and severe groups. The tree structure clusters functionally related GO terms, with node color representing adjusted *p*‐value (purple: Highly significant, yellow: Less significant) and node size representing gene count.

Based on Spearman correlation analysis, we evaluated the associations between gene expression and the abundance of all *Prevotella* species in 41 samples from Cohort II. A total of 3706 genes were identified as significantly correlated with at least one *Prevotella* species (*p* < 0.05). The 100 most significantly correlated genes are presented in a clustered heatmap (Figure 4A).

To further identify potential mediators linking *Prevotella* species to host disease, we performed differential expression analysis between the critical illness and severe groups. A total of 120 genes were significantly upregulated in the critical illness group compared to the severe group, and 15 genes were downregulated, using thresholds of |log_2_FC| > 1 and adjusted *p* < 0.05 (Figure 4B). We performed Gene Ontology (GO) enrichment analysis for biological processes. The results are visualized as a treeplot in Figure 4C. The analysis revealed a striking enrichment of immune‐related processes, which can be broadly categorized into four interconnected functional clusters: (i) immune cell activation and differentiation, including myeloid leukocyte activation, T cell differentiation, and CD4‐positive T cell activation; (ii) cytokine and inflammatory signaling, such as positive regulation of cytokine production, cytokine‐mediated signaling, and regulation of inflammatory response; (iii) immune effector functions, encompassing regulation of immune effector process, cell killing, and defense response to virus; and (iv) leukocyte migration and chemotaxis, including granulocyte chemotaxis, neutrophil migration, and leukocyte migration.

### 3.5 *P. melaninogenica* and Butyrate Pretreatment Significantly Attenuated MP‐Induced Pulmonary Inflammation

**FIGURE 5:**
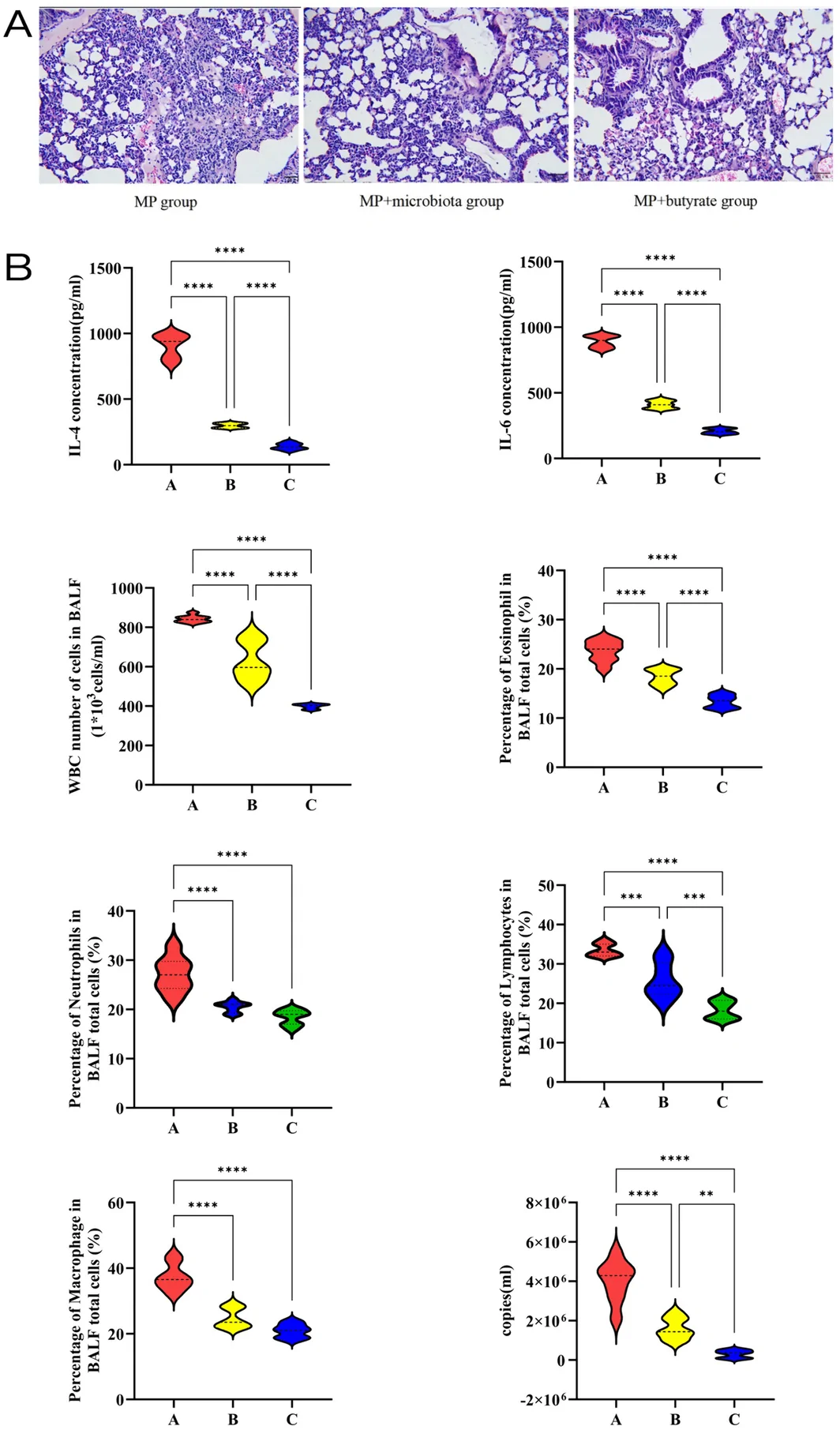
The intervention with *prevotella melaninogenica* and butyrate alleviate the inflammation of MPP. (A) Representative hematoxylin and eosin (H&E) staining of lung tissues from each group (scale bar = 100 μm). (B) Levels of IL‐6 and IL‐4 in bronchoalveolar lavage fluid (BALF), pulmonary pathogen load (copies/mL), total white blood cell (WBC) counts in BALF, and percentages of lymphocytes and LALF‐positive cells in BALF. Data are mean ± SEM. *p < 0.05 vs. MP control group (one‐way ANOVA with Tukey's post hoc test). Percentages of neutrophils, macrophages, lymphocytes, and eosinophils in BALF determined by flow cytometry. Data are mean ± SEM. *p < 0.05 vs. MP control group. A represents MP group, B represents MP + microbiota group, C represents MP + butyrate group, * represents *p* < 0.05.

Weight monitoring shows that medicine intervention can alleviate weight loss caused by inflammation, among which butyric acid intervention may be more effective than microbiota intervention (Figure S1). Histological examination using H&E staining (Figure 5A) revealed that the MP control group displayed characteristic pathological damage, including alveolar wall thickening, inflammatory cell infiltration, and interstitial edema. Notably, these histopathological alterations were less severe in the MP + microbiota group and MP + butyrate group, with lung tissue architecture resembling that of healthy controls.

Consistent with the histopathological improvements, both pretreatment strategies significantly modulated pulmonary inflammatory responses. As shown in Figure 5B, the MP control group exhibited elevated levels of the pro‐inflammatory cytokine IL‐6, which were significantly reduced in both the MP + microbiota and MP + butyrate groups (*p* < 0.05). Concurrently, levels of the anti‐inflammatory cytokine IL‐4 were also significantly decreased in both intervention groups (*p* < 0.05), indicating an overall attenuation of the inflammatory state. In parallel, pathogen load decreased progressively across the MP, MP + microbiota, and MP + butyrate groups (*p* < 0.05). Compared to the MP group, the two intervention groups also showed significant reductions in total white blood cell counts, lymphocyte percentages, and LALF‐positive cell percentages in bronchoalveolar lavage fluid (all *p* < 0.05). Collectively, these findings demonstrate that both pretreatment strategies control infection and alleviate local immune‐inflammatory infiltration, with the MP + butyrate group exhibiting a more pronounced therapeutic effect.

## 4 Disscussion

Metagenomic pathway profiling revealed multiple differentially abundant metabolic pathways between the critically ill and severe groups, with butanoate metabolism significantly enriched in the severe group. This is similar to a research finding (Wang et al. 2024). Correlation analysis further demonstrated that the abundance of Prevotella species exhibited significant correlations with multiple metabolic pathways. Notably, the abundance of most Prevotella species was positively correlated with the butanoate metabolism pathway, as well as with several other metabolic pathways. Conversely, Prevotella abundance was negatively correlated with multiple pro‐inflammatory pathways, including lipopolysaccharide biosynthesis, linoleic acid metabolism, and tryptophan metabolism. These are similar to some studies' findings recently (Larsen 2017; Gao et al. 2025; Nieman et al. 2025; Schenkein et al. 2020). These results indicate that *Prevotella* enrichment is closely associated with enhanced anti‐inflammatory metabolic potential and reduced capacity for pro‐inflammatory metabolism (Yu et al. 2025; Peng et al. 2024; Jiang et al. 2022; Scher et al. 2013). Based on these findings, we hypothesize that *P. melaninogenica* may exert local anti‐inflammatory effects through the production of butyrate (Chen et al. 2024) and other anti‐inflammatory metabolites (Li et al. 2025; Mann et al. 2024; Huang et al. 2023).

Correlation analysis identified a large set of host genes significantly correlated with *Prevotella* species (Priya et al. 2022). Differential expression analysis between severity groups further identified a set of differentially expressed genes that overlapped with *Prevotella*‐associated genes (Ju et al. 2025). Functional enrichment analysis revealed that these overlapping genes were significantly enriched in immune‐related biological processes (Zhang et al. 2025). These findings suggest a broad immunomodulatory role of the genus *Prevotella* in MPP.

Animal experiment results directly demonstrate the protective potential of *P. melaninogenica* , as a representative species of the genus *Prevotella*, in MPP. More importantly, direct butyrate supplementation showed protective effects across all evaluated parameters (Misharin et al. 2013; Fan et al. 2024). This strongly suggests that butyrate is a key effector molecule mediating the protective effects of the genus *Prevotella* (Lei et al. 2025). In the context of severe microbial dysbiosis characteristic of critical MPP, directly supplementing the downstream effector may be more straightforward and effective than attempting to restore specific upstream commensals (Liu et al. 2023, 2018; Rob et al. 2025).

However, several limitations should be acknowledged. First, our proposed mechanistic model of the genus *Prevotella* exerting anti‐inflammatory effects through butyrate is primarily based on correlation analyses and literature inference (Gianicolo et al. 2020). Future in vitro cellular experiments and gene knockout mouse models are needed for rigorous causal validation of the mechanisms involving *P. melaninogenica* and its metabolite butyrate (Wang et al. 2023). Second, the optimal intervention timing, dosage, and administration frequency for *P. melaninogenica* and butyrate remain to be optimized through systematic animal studies (Shen et al. 2024).

In conclusion, through integrated multi‐omics analysis and animal models, this study reveals respiratory microbiota dysbiosis is associated with MPP severity. *Prevotella melaninogenica* may alleviate airway inflammation through its metabolite butyrate.

## Author Contributions

**Zhengyang Xue:** conceptualization, investigation, writing – original draft, writing – review and editing, visualization, validation, methodology, formal analysis, project administration, software, data curation, supervision, resources. **Huiliang Xu:** investigation, conceptualization, software, validation. **Ling Zhu:** validation, writing – review and editing, formal analysis, supervision. **DeYu Zhao:** resources, writing – review and editing, writing – original draft, visualization.

## Funding

The authors have nothing to report.

## Ethics Statement

This study was approved by the research ethics committee of the authors' institution (approval numbers: 202012089‐1, 02211213‐1, WXCH2022‐12‐106, 2023‐018) and complied with the Declaration of Helsinki.

## Consent

The authors have nothing to report.

## Conflicts of Interest

The authors declare no conflicts of interest.

## Supporting information


**Figure S1:** Daily weight changes of mice. * represents *p* < 0.05.

## Data Availability

The data that support the findings of this study are available on request from the corresponding author. The data are not publicly available due to privacy or ethical restrictions.
