# Supplementary material for: Prevotella melaninogenica Alleviate Mycoplasma pneumoniae Infection Through the Butyrate Based on Multi‐Omic Analysis and Experimental Validation
Source: Chem Biol Drug Des. 2026 Sep 6;108(3):e70397. doi: 10.1111/cbdd.70397 (PMC13547638; doi:10.1111/cbdd.70397)
Supplement: Supplementary file 1 [file CBDD-108-e70397-s001.docx]

# Supplementary Materials

# Supplementary Figure 1. Daily weight changes of mice. * represents *P* <0.05.
